# Assessment of Respiration-Induced Motion and Its Impact on Treatment Outcome for Lung Cancer

**DOI:** 10.1155/2013/872739

**Published:** 2013-06-04

**Authors:** Yan Wang, Yong Bao, Li Zhang, Wei Fan, Han He, Zong-Wen Sun, Xiao Hu, Shao-Min Huang, Ming Chen, Xiao-Wu Deng

**Affiliations:** ^1^Department of Radiation Oncology, State Key Laboratory of Oncology in Southern China, Cancer Center, Sun Yat-Sen University, Guangzhou, Guangdong 510060, China; ^2^Department of Radiation Physics, Cancer Center, Sun Yat-Sen University, Guangzhou 510060, China; ^3^Department of Nuclear Physics, Cancer Center, Sun Yat-Sen University, Guangzhou 510060, China; ^4^Department of Radiation Oncology, The First People's Hospital of Foshan City, Foshan 528800, China; ^5^Department of Radiation Oncology, The First People's Hospital of Jining City, Jining 272011, China

## Abstract

This study presented the analysis of free-breathing lung tumor motion characteristics using GE 4DCT and Varian RPM systems. Tumor respiratory movement was found to be associated with GTV size, the superior-inferior tumor location in the lung, and the attachment degree to rigid structure (e.g., chest wall, vertebrae, or mediastinum), with tumor location being the most important factor among the other two. Improved outcomes in survival and local control of 43 lung cancer patients were also reported. Consideration of respiration-induced motion based on 4DCT for lung cancer yields individualized margin and more accurate and safe target coverage and thus can potentially improve treatment outcome.

## 1. Introduction

Respiration-induced tumor motion is a significant source of geometric uncertainty in radiotherapy for thoracic malignancies [1, 2]. In the era of three-dimensional (3D) conformal radiotherapy, a tumor is usually imaged at a random time point by a free-breathing CT and encompassed by a generalized empirical respiratory-motion margin. However, in free-breathing CT, a thoracic structure can be distorted due to the motion, resulting in either lengthening or shortening of the structure in the motion direction [3]. Because of such motion artifact, the size, shape, and position of moving tumor or other organs cannot be depicted accurately. Furthermore, the conventionally used “standard safety margins” are defined without explicit measurements of the individual tumor motion [4, 5]. Recent studies show that individual, as opposed to a standard population-based margin, is essential for high-precision radiotherapy of lung cancer [2, 6–9].

Adding the fourth dimension, time, to three-dimensional CT is termed four-dimensional CT (4DCT) [10, 11]. 4DCT image data provides important spatial and temporal information including the entire range of moving targets and organs during quiet respiration [12] and has been used to characterize intrafractional respiration mobility and to determine an individualized tighter margin around the target [13]. The improved geometric accuracy would increase the therapeutic gain as it allows escalated dose to the tumor and/or improved sparing for healthy tissue [9].

Studies on the assessment of lung tumor motion using different approaches, which ranged from fluoroscopy [14], to orthogonal portal films [15], dynamic magnetic resonance imaging (MRI) [16], slow CT [7], breath-hold CT [17], and 4DCT [18–20], have been reported. The tumor motion was assessed with implanted gold markers in or near tumor [15] or by the centroid or border of tumor [7, 14, 16–21]. It was observed that the closer the tumor to the diaphragm, the more mobile the tumor was [7, 15, 16, 18–21], because the respiration was primarily driven by the diaphragm motion. In vast majority of studies, small solitary tumors showed larger motions than bigger ones [15, 20], and the magnitude of tumor motion was always the greatest in the superior-inferior (SI) direction [7, 15, 16, 18–21].

The 4DCT is proven to be more effective and objective for the evaluation of breathing motion. This is still of great interest for more comprehensive understanding on factors associated with respiration-induced tumor motion and on its impact of treatment outcome. The main purpose of this work is, then, to determining these factors and the impact by analyzing the respiration motion and outcome data collected for 4DCT-based radiotherapy in our clinic.

## 2. Materials and Methods

### 2.1. Ethics Statement

This retrospective research was conducted according to the principle described in the *Declaration of Helsinki*. The research protocol was reviewed and approved by Ethical Review Committee of Sun Yat-Sen University Cancer Center (SYSUCC) (approval number, YP2008032).

### 2.2. Study Patients

After a review of medical records in our hospital, we identified 43 patients with 44 lung tumors who underwent 4DCT scans during quiet respiration between September 2005 and January 2008. Each patient had at least one pulmonary lesion with distinct boundary. Tumor staging was done by contrast-agent CT chest and abdomen, magnetic resonance imaging (MRI) of the brain, and bone scintigraphy whereas positron-emission tomography and CT were not mandatory. All patients who had local and/or regional disease, received a curative intent radiotherapy with or without chemotherapy. Karnofsky Performance Status (KPS) ≥70 and weight loss <5% in half a year were required. Exclusion criteria included previous thoracic surgery, previous radiation treatment, clinically significant pleural effusion limiting delineation of the total extent of the primary tumor, lobar atelectasis, and an inability to breathe in a reproducible manner (breath variability was more than 5%). Patients were excluded from the study in survival analysis section if their total dose was <60 Gy. 

### 2.3. 4DCT Procedure

At SYSUCC, a 16 slice 4DCT scanner (GE Lightspeed, GE) with a respiration management system (RPM, Varian) was used to acquire respiration correlated CT. Retrospective 4DCT scanning entailed the generation of multiple slices at each relevant table position, during at least the length of a full respiration cycle (oversampling). The acquired data (about 1000 images) were sorted into 10 datasets correlating with 10 phases of the respiratory cycle. The phase 0% represents the end of inspiration, with the phase 50% for the end of expiration. The spatial resolution along the superior-inferior direction was limited by the 2.5 mm slice thickness.

### 2.4. Tumor Delineation and Motion Measurement

The gross tumor volumes (GTVs) on ten respiratory phases were delineated using a treatment planning system (Pinnacle^3^, version 7.6c, Philips). All GTVs were delineated with an autosegmentation tool using threshold −750 to 4096 Hu first then manually modified by a single radiation oncologist. All contours were checked by two other radiation oncologists for consistency.

The GTV volumes for the ten phases were recorded as GTV-0%, GTV-20%,…, and GTV-90%. The mean GTV volume was calculated from the average GTVs of ten respiratory phases as mean GTV = 1/10 (GTV-0% + GTV-10% + *⋯* + GTV-90%). Internal gross tumor volume (IGTV) was obtained by combining the GTVs in ten phases of the respiratory cycle, that is, by the union of the 10 GTVs.

The centriods of GTVs in ten phases were determined by the planning system and were used to calculate the magnitudes of motions in three directions termed as dLR, dAP, and dSI, the distance between the two extreme positions in left-right (LR), anterior-posterior (AP), and superior-inferior (SI) direction during the respiratory cycle phases, respectively. The 3D vector was calculated as follows: 3D vector = $\sqrt{{d\text{LR}}^{2}+{d\text{AP}}^{2}+{d\text{SI}}^{2}}$. Program MATLAB 7.4 (MathWorks) was used to compute and plot 3D point trajectories.

The CT set at 20% phase was chosen as reference CT to determine the relative GTV location in the lung. The location parameter consisted of three directional components, fLR, fAP, and fSI, corresponding to the relative fractional location in the lung in LR, AP, and SI directions, respectively. For example, fSI was the distance between the apex of the lung and the GTV centroid divided by the distance between the apex and the diaphragm point passing through the GTV centroid in the SI direction. For the LR and AP directions, the same method was applied except that the distance was defined from the centriod to the carina (for LR) or to the anterior boundary of the lung (for AP), and the divisor was defined as ipsilateral lung LR or AP diameter. The attachment degree to rigid structure (e.g., chest wall, vertebrae, or mediastinum, which all minimal respiratory motion) was defined as the ratio of the longest diameter attached to the rigid structure divided by longest diameter of the tumor in transversal plane. 

### 2.5. Treatment Planning and Delivery

 In the clinic, GTVs included the primary tumor (GTV-T), positive lymph nodes (GTV-N) with lymph nodes in the mediastinum with a short diameter >1 cm, or lymph nodes with positive tumor cell sampling, or clusters of small lymph nodes of short diameter <1 cm within 1 region, or ^18^F-FDG standard uptake value >2.5 on PET/CT at initial staging. IGTV was obtained on 4DCT maximal intensity projection or ten phases. For patients who had squamous cell carcinoma, the clinical target volume tumor (CTV-T) included IGTV-T with a margin of 0.6 cm. For patients who had adenocarcinoma or histology not otherwise specified nonsmall cell lung cancer (NSCLC) or small cell lung cancer (SCLC), the CTV-T was created by IGTV-T with a margin of 0.8 cm. The clinical target volume node (CTV-N) included the positive lymph nodes region only. A 5 mm expansion uniformly around the CTV created the planning target volumes (PTV).

6–8 MV X-rays were used. All NSCLC patients underwent radiotherapy with conventional fractionation schemes. Tumors were prescribed as high as possible (not lower than 60 Gy) based on normal tissue dose-volume constraint. For locally advanced NSCLC (T3-4NxM0 or TxN2-3M0) patients, concurrent chemotherapy consisted of weekly or 2 cycles of monthly cisplatin and taxane-based regimens. For limited-stage SCLC patients, thoracic radiotherapy was administrated with a total dose of 45 Gy and at hyperfractionated technique of 1.5 Gy/fraction twice daily. The minimal interval between fractions was 6 hours. Patients received thoracic radiotherapy within the first 2 cycles of cisplatin and etoposide (EP). Patients who achieved complete remission (CR) or partial remission (PR) of tumor after the completion of chemoradiotherapy (4–6 cycles of EP plus concurrent thoracic radiotherapy) were offered prophylactic cranial irradiation (PCI), which was delivered daily to a total dose of 30 Gy over a period of 3 weeks or 25 Gy over 2 weeks.

### 2.6. Followup

After completion of treatment, patients were reviewed within 4–6 weeks, then every 3 months in the first 2 years, and every 4 months in the third year, every 6 months thereafter. Physical examination and CT scans of the thorax and upper abdomen were performed routinely. 

### 2.7. Statistical Analysis

SPSS 13.0 statistical software was used (SPSS Inc., Chicago, IL). To understand what factors could be associated with and predictive of tumor motion, logistic regression (backward stepwise method) was used to test the relationship between GTV motion and clinical or anatomic factors, which were either continuous or categoric variables (e.g., gender, histology, age, GTV volume, tumor location, and attachment degree to the rigid structure). According to the results of the statistical analysis and observation of the GTV motion pattern, we calculated *R* ^2^ values to assess the possible correlation between the GTV centroid 3D vector motion and the relevant factors. Actuarial overall survival (OS), cancer-specific survival (CSS), progression-free survival (PFS), local progression-free survival, and distant metastasis-free rate were estimated by the Kaplan-Meier model, and the significances were tested by log-rank. The time for survival or failure was calculated from the first day of treatment intervention. 

## 3. Results

### 3.1. Patient and Treatment Characteristics

Patient characteristics are listed in Table 1. The median age of the 38 men and 5 women was 56 years (range: 35–78 years). Twenty-seven patients had NSCLC (Stage I in 1, II in 1, IIIA in 5, IIIB in 15, and IV in 5), fifteen had limited-stage SCLC, and one had nasopharyngeal carcinoma with isolated lung metastases. The most common involved lobes were the upper lobes (36.4% left and 38.6% right). The median GTV was 45 cm^3^ (range, 0.5–454 cm^3^), and the median radiation dose of NSCLC was 62 Gy given in 31 fractions (range: 54–70 Gy, 2 Gy per fraction), one fraction daily. All SCLC patients received thoracic radiotherapy of 45 Gy with 1.5 Gy twice a day in 30 fractions. Most patients (39, or 82.9%) had received concurrent chemotherapy (CCRT), and the most common concurrent chemotherapy regimen was paclitaxel (45–50 mg/m^2^ weekly). 

### 3.2. GTV Centroid Movement

**Figure 1:**
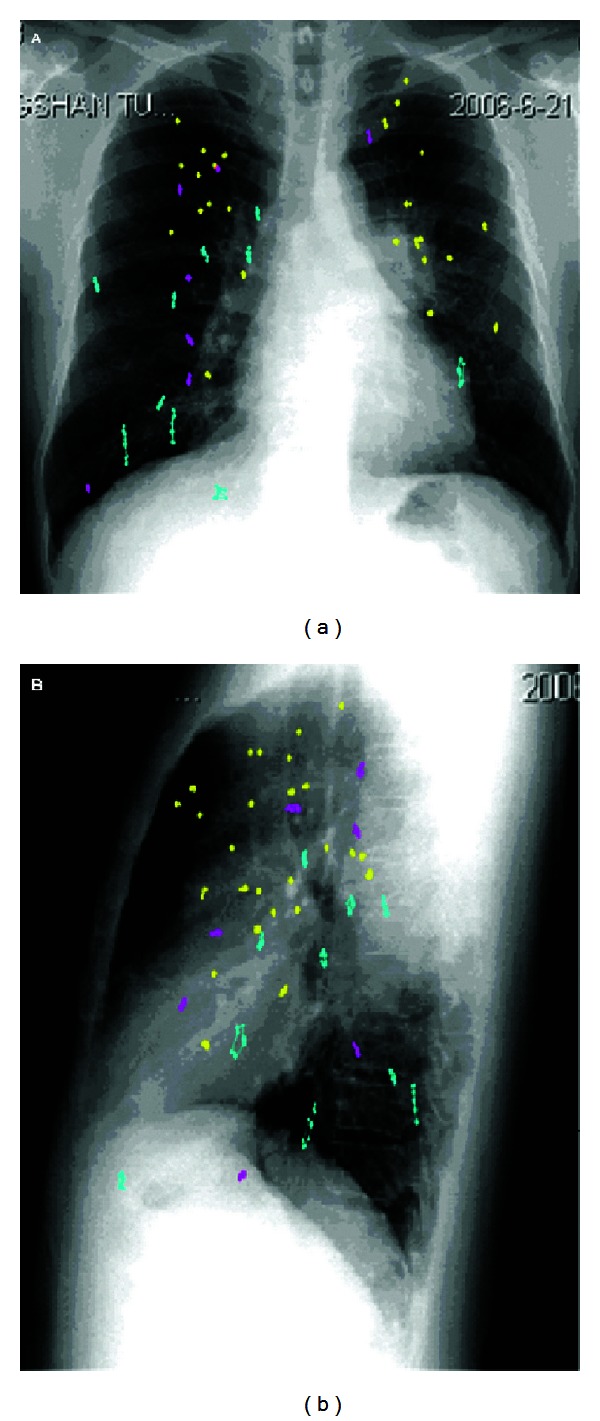
The 3D point trajectories of GTV centroid. Green plots represent the movement of tumor center of mass is more than 5 mm in any directions (10/44), yellow points mean whose movement is less than 3 mm in three directions (27/44), and red plots represent whose is movement between 3–5 mm (7/44). (a) anterior-posterior view; (b) lateral view.

GTV centroid motion exceeding 5 mm was seen in 10 of 43 patients (23%), while 61% of lung tumors moving less than 3 mm (Figure 1). Maximum magnitude of the tumor centroid of 5.3 mm, 5.2 mm, and 14.4 mm was observed in the LR, AP, and SI direction, respectively. Averaged over all patients, the means and 1SD of the LR, AP, and SI motion were 1.2 ± 0.9 mm, 1.6 ± 1.1 mm, and 2.9 ± 3.4 mm, respectively. The maximum expected motion with a 95% percentile about the centroid of GTV for LR, AP, and SI direction was 2.6 mm, 4.8 mm, and 13.05 mm, respectively.

**Figure 2:**
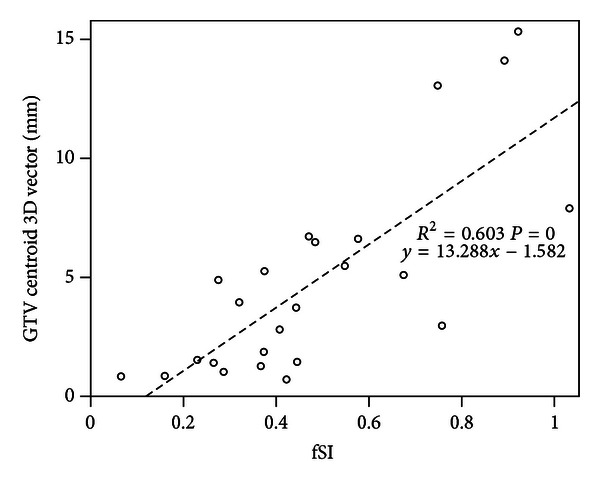
Relationship between vector and fSI, filtered out attachment degree to rigid structure >0.6, *y* = 13.288*x* − 1.582*R* ^2^ = 0.603 Abbreviation: fSI: fractional superior-inferior location; 3D vector = $\sqrt{d\text{L}{\text{R}}^{2}+d\text{A}{\text{P}}^{2}+d\text{S}{\text{I}}^{2}}$.

Analysis of all data revealed that the variations in GTV centroid 3D vector movement was associated with GTV size, the SI tumor location in the lung, and the attachment degree to a rigid structure, such as chest wall, vertebrae, or mediastinum (Table 2). Nonlinear relationship was observed between vector displacement and GTV volume or length, and so was the attachment degree to rigid structure. There was a weak positive correlation between centroid vector mobility and fSI, implying that patients with lower lesion disease tend to exhibit larger tumor movements. Excluding the cases with attachment degree more than 0.6, the correlation would enhance with a correlation coefficient (*R*) from 0.418 to 0.603 (Figure 2).

The peripheral lung tumor located near the diaphragm showed the greatest degree of motion, followed by upper-lobe posterior-segment solitary tumors. Detailed characteristics of high-mobility tumors are summarized in Table 3. The dominant displacement was in SI and/or AP directions. The rotational motions of lower lobe tumors were observed.  Table 4 showed the magnitude of GTV centroid mobility with different positional and attachment status. 

The more attached to the rigid structure, the less mobile of tumor was. For those tumors located to the mediastinum, movement in AP direction was a major contributor to the GTV motion, with the magnitude less than 3.5, 4, and 3.5 mm in LR, AP, and SI direction. These movements were probably associated with cardiac contraction and/or aortic pulsation. 

In all 20 patients with an attachment degree to rigid structure of being more than 0.6, the magnitude was small, with 1.0 ± 0.6, 1.5 ± 0.9, and 1.5 ± 1.5 mm in the LR, AP, and SI directions, respectively. For this tumor group, there were 2 outliers with large motion in SI direction, with the magnitude of 5.1 mm and 4.5 mm. One located in lower lobe, the other in upper lobe but posterior segment. When filtered out the outliers, maximum movement observed in the LR, AP, and SI directions were 2 mm, 3.7 mm, and 2.8 mm.

For big tumor (GTV ≥45cm^3^) located upper 1/3 and middle 1/3, the magnitude in AP direction was dominant. For small tumor (GTV <45cm^3^), the largest motion was in SI direction. 

### 3.3. Survival

**Figure 3:**
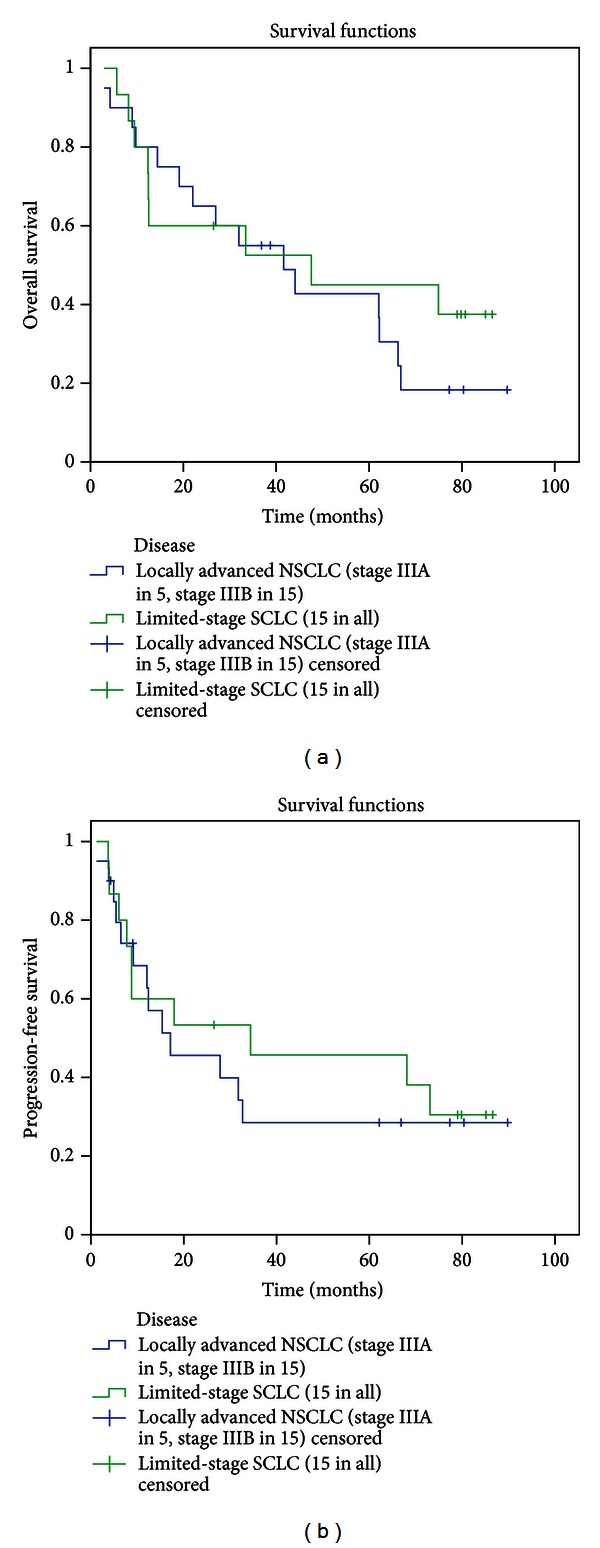
Overall survival (a) and progression-free survival curves (b) of local advanced NSCLC and limited-stage SCLC.

Up to February 2013, the median follow-up duration was 32.6 months (range, 1.9–89.8 months) in all patients; 80.4 months (range: 65.6–89.8 months) in the survivors: and 20.6 months (range: 3.0–75.0 months) in patients who had died. Four NSCLC patients (Stage I in 1, IIIB in 2, and IV in 1) and four limited-stage SCLC patients are alive and free of disease. One SCLC patients is alive with local disease recurrence underwent salvage treatment. Twelve patients have died of distant metastases (NSCLC in 8, SCLC in 4). Eight patients have died of locoregional progression inside the thorax and metastases both (NSCLC in 5, SCLC in 3). One SCLC patient died of local progression inside the radiation field. Two NSCLC patients have died of treatment-related toxicity. One NSCLC patient has died with the metastases for secondary cancer in rectum. Two patients have died as a result of other medical conditions, one for the cause of sputum jams and other for severe pneumonia. For NSCLC subgroup (Stage IIIA in 15 and IIIB in 5), the median survival time was 41.6 months. At 1-, 3- and 5-year actuarial survival was 75%, 55%, and 36.7%, whereas SCLC patients (limited-stage in 15) have a 1-, 3, and 5-year survival of 73.3%, 52.5%, and 37.5%, respectively, with the median survival time of 47.6 month (Figure 3(a)). One advanced NSCLC patient with cervical lymph node metastases who accepted definitive chemoradiotherapy has an overall survival time without progress of 89.8 months by the time of followup. At 1, 3, and 5 years, cancer-specific survival was 84.4%, 58.1%, 37.6% for Stage III NSCLC and 73.3%, 52.5%, and 45% for limited-stage SCLC, respectively.

### 3.4. Patterns of Treatment Failure

**Figure 4:**
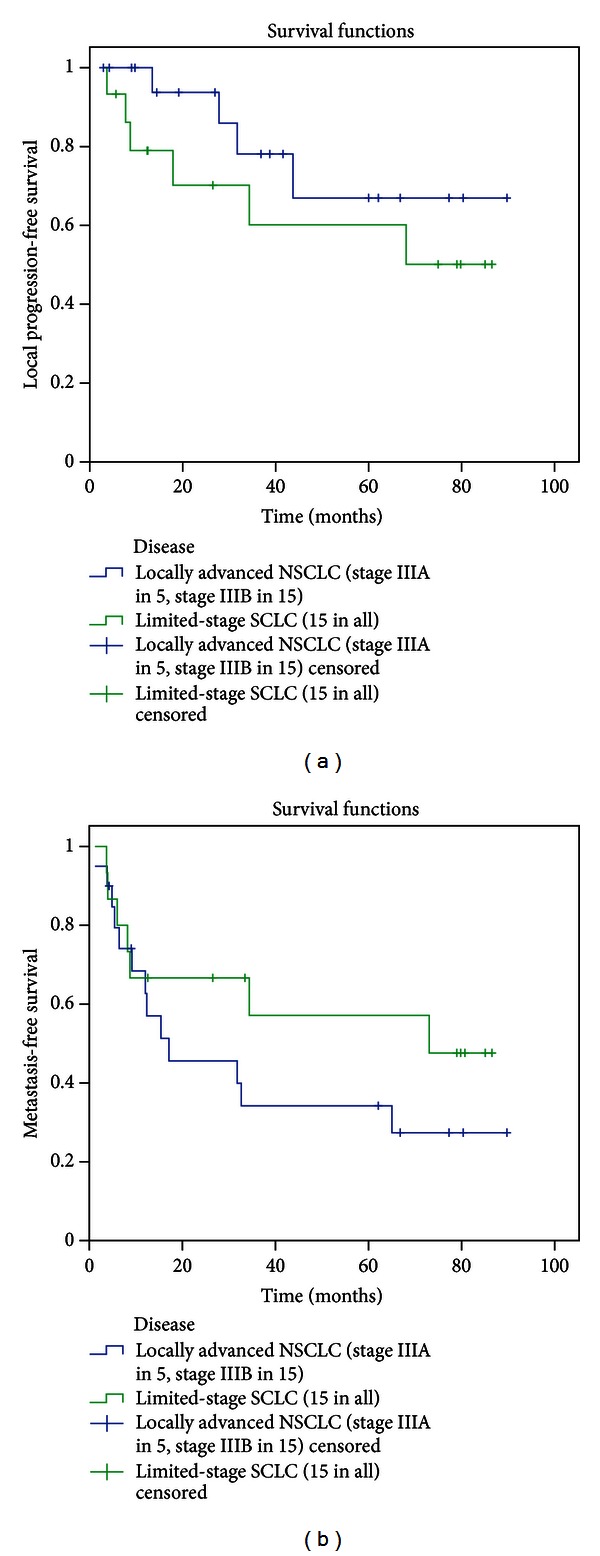
The local progression-free survival (a) and metastasis-free survival curve (b).

The median progression-free survival time was 17.1 months for NSCLC group and 34.4 months for SCLC group; 1, 3-year progression-free survival were 62.7% versus 60% and 28.5% versus 45.7% for NSCLC group and SCLC group (Figure 3(b)). Actuarial local progression-free survival at 1 and 3 years was 93.8% and 67% for NSCLC group and 79% and 60.2% for SCLC group, respectively (Figure 4(a)). The 1-, 3- and 5-year metastasis-free survival rate was 62.7%, 34.2%, and 27.4% for NSCLC and 66.7%, 57.1%, and 47.6% for SCLC, respectively (Figure 4(b)).

Of the 25 patients who experienced treatment failure or died, 4 patients (NSCLC in 1, SCLC in 3) developed local and/or regional tumor progression without distant metastases; 14 patients (NSCLC in 10, SCLC in 4) developed metastatic disease without locoregional progression; and 7 patients (NSCLC in 4, SCLC in 3) showed concurrent thoracic and distant metastatic progression during the follow-up phase.

## 4. Discussion

The reliability of motion results in this work was dependent on two factors, regularity in patient breathing and consistency in GTV delineation. Data from both phantoms and clinical practice demonstrated that respiratory regularity was of most importance to reduce motion artifacts during 4DCT scan [22, 23]. In our research, all patients underwent breathing training and those with breathing variability >5% were eliminated from the study. Delineation uncertainty certainly existed [24]. In order to minimize factitious factors during the delineation of GTV, we used an auto-segmentation tool with a fixed threshold and a selected window width and level. So-obtained GTVs were manually modified by a single radiation oncologist to reduce interobserver variability [25]. Before analysis, all contours were inspected independently by two radiation oncologists to ensure high contouring consistency.

It was found that tumor centroids vector movement was associated with GTV size, the SI tumor location (fSI), and the attachment degree to rigid structure. This result was intuitive but contrasted to the studies by Stevens et al. [6] and van Sörnsen et al.[7] in the early 2000s. Both studies reported that lung tumor motion was independent and cannot be predicted by any factors. Possible cause of this discrepancy included limited number of cases (the former in 22, the latter in 29), and different assessment approaches were used (the former used orthogonal radiographs; the latter was based on slow-CT images). In another study of 166 lung tumors based on 4DCT, Liu et al. [19] reported a significant correlation between tumor motion and diaphragm motion, the SI tumor location in the lung, GTV size, and disease T stage. But the related factors were not independent variables required questionable. For example, T stage sometimes included the surgery difficulty information caused by GTV size or tumor location. In the present study, our analysis revealed there was a weak positive correlation between tumor motion and the SI tumor location, but the relationship of tumor motion and GTV size or attachment status was not linear. We inferred it was because the related factors possibly had a so-call “interaction effect” and could influence each other, though they were looked at independently in our study.

Similar to the previously published studies [18, 19], besides tumors located in lower lobe, tumors in posterior side of the lung exhibited greater mobility only ranking second. The reason Maxim et al. [18] implied was that the lower lobes occupied a large proportion of the posterior thorax. Liu et al. [19] inferred that the diaphragm can exhibit a large degree of rotational movement. And another possibility appear to be that the diaphragm motion in posterior side was more moveable than that in anterior side; even upper lobe could be affected. No significant correlation was observed between centriod vector and the location in AP direction, both in our and published studies. It might partly due to small sample size. 

Of the three related factors, tumor location probably weighted more than GTV size and attachment degree to rigid structure. This is supported by our original findings as follows. Firstly, the peripheral lung tumors located near the diaphragm showed the greatest degree of motion, followed by upper-lobe posterior-segment solitary tumors. Even in the tumors with an attachment degree to rigid structure of more than 0.6, which estimated very small magnitude, still can be seen cases in these two location moved more than 5 mm. Secondly, when excluding all the cases with attachment degree more than 0.6, the linear correlation between centroid vector mobility and fSI would enhance, regardless of tumor size. Thirdly, regardless of tumor attachment status, for big tumors (volume ≥45 cm^3^) located upper 1/3 and middle 1/3 and those close to the mediastinum, magnitude in AP direction was dominant. Those results indicated that more consideration should focus on tumor location when determining internal margin for target mobility.

Compared with our study, Liu et al. [19] reported a higher incidence of large GTV displacements in 166 lung tumors. For 95% tumors, the magnitude of motion was less than 13.4 mm, 4 mm, and 5.9 mm in SI, LR and AP directions, respectively. In our study, the corresponding values were 13.05 mm, 2.6 mm, and 4.8 mm. In their study the proportions of tumors that moved >5 mm in SI, LR, and AP direction were 39.2%, 1.8%, and 5.4%, respectively. In our study, the corresponding percentages were 22.7%, 2.3%, and 2.3%. Two possible reasons for the slight discrepancy were the less distribution of tumor located in middle or lower lobe (25%) and the high proportion tumors attached to rigid structure (68.2%) in our study.

4DCT simulation can provide benefits in selected patients. Similar to that reported by Rietzel et al. [4]; the use of 4DCT resulted in significantly smaller and safe PTV margins than those derived using conventional 3DCT scans and the “standard” margin. It will be of clinical significance when administrating a dose to a bulky tumor, especially those limited by pulmonary or spinal toxicity without 4DCT. The smaller PTVs with 4DCT would result in less normal tissue irradiated with higher dose, thus, transforming a palliative intent to a curative one with toxicity at tolerable level. On the other hand, modern high-precision radiation delivery requires accurate knowledge for issues such as internal motion and setup. For example, the use of individualized PTV margins based on 4DCT would be essential for hypofractionated or stereotactic radiotherapy, as pointed out by Keall et al. [11].

The predominant cause of deaths for lung cancer is believed to be distant metastases and local recurrence. Local failure remains a major challenge when treating lung cancer with radiotherapy, as high as 30%–50% recurrence rate at 5 years in NSCLC [26] and 36%–52% in SCLC [27]. Animal experiments and clinical data in lung cancers indicated that improvements in local control would decrease distant metastases, as a part of the distant metastases was derived from local recurrences of the cancer [28]. 

In this work, we observed unexpected and promising local control and survival rate with the use of 4DCT for both NSCLC and SCLC. The 5-year overall survival rates of 36.7% for NSCLC and 37.5% for SCLC were encouraging undoubtedly, as compared to the 5-year overall survival rate of around 20% from conventional treatments for locally advanced NSCLC and limited-stage SCLC as reported in the literature and in our own clinical experience [27, 29]. Though the statistical power of this result is not strong enough to draw a firm conclusion, the positive effect of 4DCT result in accurate and safe target coverage should be given enough attention. It has the potential to improve treatment outcome. It is also believed that the geometric precision is only one of the key factors of the gain in survival and local control, but never the only one. Several other factors may contribute to clinical outcome in this study. (1) All patients with curative intent belonged to the “favorable group” (KPS ≥70, weight loss <5% in half a year). (2) The CCRT for locally advanced NSCLC and EP chemotherapy plus thoracic radiotherapy for limited-stage SCLC were the principle part of treatment. According to the recently meta-analyses [29], the CCRT itself can decrease locoregional progression and improve overall survival in NSCLC. (3) The start of thoracic radiotherapy in the limited-stage SCLC patients was all within 2 cycles of chemotherapy in our study, which was proved to be favorable in survival than the late start of radiotherapy [30]. 

Liao et al. [31] reported 91 NSCLC patients underwent CCRT with 4DCT/IMRT. The median survival times were 16.8 months for the 4DCT/IMRT group. From the survival curve, 1-year free of local-regional progression was about 87%, 1-year free of distant metastase was about 60%, and 1-year overall survival was about 70%, which were in good accordance with our study. To our knowledge, there is no study reporting survival benefit with using 4DCT for SCLC.

Although with the use of 4DCT, we can safely reduce the margin to account for intrafractional respiration motion, other components contributing to PTV margin, such as interfractional variations, set-up margin, need to be considered. While every effort was made to keep breath regular and ensure target delineation accurate, some artifacts and inconsistency were hard to eliminate. The limited number of cases analyzed is the major drawback of the current study. Our ongoing research is to increase number of the cases and to update the results in the future.

## 5. Conclusion

The 4DCT data in this work indicate that the peripheral lung tumors located near the diaphragm show the greatest degree of respiration motion, followed by upper-lobe posterior-segment solitary tumors. Tumor respiration motion was found to be associated with tumor location, volume, and attachment to rigid structures, with tumor location being the most important factor among the other two. The use of 4DCT resulted in the use of individualized margin to account for patient-specific breathing motion, improving the accuracy for tumor targeting during radiotherapy. This may contribute to the improved local control and overall survival as observed presently for both NSCLC and SCLC. 

## Figures and Tables

**Table 1:** Patient and treatment characteristics of the 43 patients.

Factors	Characteristic	Number of cases	Percentage
Gender	Male	38	88.4%
	Female	5	11.6%
Age (years old)	Median	56	
	Range	35–78	
	NSCLC	27	62.8%
Histology	SCLC	15	34.9%
	Metastases	1	2.3%
	I-II	2	7.4%
Stage of NSCLC (*n* = 27)	IIIA	5	18.5%
	IIIB	15	55.6%
	IV	5	18.5%
Stage of SCLC (*n* = 15)	Limited stage	15	100%
	T1	10	23.8%
T status*	T2	7	16.7%
	T3	7	16.7%
	T4	18	42.8%
	N0-1	5	11.9%
N status*	N2	16	38.1%
	N3	21	50%
	Left upper lobe	16	36.4%
	Left lower lobe	1	2.3%
Tumor location^†^	Right upper lobe	17	38.6%
	Right middle lobe	4	9.1%
	Right lower lobe	6	13.6%
GTV volume (cm^3^)	Median	45	
	Range	0.5–454	
Tumor attachment status^†^	Solitary tumor	14	31.8%
	Attached tumor	30	68.2%
	Median	62	
Treatment dose of NSCLC (Gy) (*n* = 27)	Range	54–70	
	<60	6	22.2%
	60–65	13	48.1%
	66–70	8	29.6%
Treatment dose of SCLC (*n* = 15)	45	15	100%
Concurrent chemoradiotherapy*	NSCLC	24^‡^	88.9%
	SCLC	15	100%

*One nasopharyngeal carcinoma patient with isolated lung metastases was not included in the T or N status calculation.

^†^One NSCLC patient had two lung lesions.

^‡^Two early patients received radiation alone. One locally advanced patient canceled chemotherapy for active tuberculosis.

NSCLC: nonsmall cell lung cancer; SCLC: small cell lung cancer; GTV: gross tumor volume.

**Table 2:** Relationship of GTV centroid 3D vector with clinical and anatomic factors by logistic regression.

Clinical and anatomic factors	*P* value
Gender	0.198
Age	0.095
Histology	0.114
fLR	0.073
fAP	0.111
fSI	0.001
GTV volume (cm^3^)	0.046
Attachment degree to rigid structure	0.008

Logistic regression suggested that the tumor centriod 3D vector was associated with GTV volume, fSI (the superior-inferior tumor location in the lung) and the attachment degree to rigid structure (e.g., chest wall, vertebrae, or mediastinum).

NSCLC: nonsmall cell lung cancer; SCLC: small cell lung cancer; fLR, fAP, fSI: fractional left-right, anterior-posterior, and superior-inferior location, respectively; GTV: gross tumor volume; attachment degree to rigid structure: the ratio of the longest diameter attached to the rigid structure divided by longest diameter of the tumor in transversal plane.

**Table 3:** The characteristics of high-mobility tumors (movement more than 5 mm).

Case	Tumor location	GTV (cm^3^)	Longest diameter (cm)	Attachment degree	fAP	fSI	dLR (mm)	dAP (mm)	dSI (mm)	Vector (mm)
A	RUL (apicoposterior segmental)	24.9	4.6	0.5	0.56	0.37	0.8	1	5.1	5.3
B	RUL (posterior basal segmental)	31.6	5.9	0.47	0.68	0.47	2.5	2.3	5.8	6.7
C	RUL (posterior basal segmental)	2.2	1.6	0	0.46	0.54	1.4	1	5.2	5.5
D	RUL (posterior basal segmental)	2.4	1.9	0	0.61	0.57	0.7	1.9	6.3	6.6
E	RLL (lateral posterior basal segmental)	165.3	6.2	0.92	0.78	0.82	2.5	2.1	5.1	6.1
F	RLL (dorsal segmental)	0.55	1.2	0	0.76	0.51	1.4	2	6	6.5
G	LLL (lingular bronchus)	12.5	2.9	0	0.42	0.74	2.6	5.2	11.7	13.1
H	RLL (anteriorbasal segmental)	2.2	1.4	Attach to diaphragm	0.12	0.9	5.3	1.7	5.6	7.9
I	LLL (lateral posterior basal segmental)	59.6	4.9	Attach to diaphragm	0.83	0.89	1.3	1.3	13.5	14
J	RLL (lateral basal segmental)	17.0	3.7	Attach to diaphragm	0.58	0.92	1.9	4.9	14.4	15.3

GTV: gross tumor volume; RUL: right upper lobe; RLL: right lower lobe; LLL: left lower lobe; fAP, fSI: fractional anterior-posterior, superior-inferior location, respectively; dLR, dAP, and dSI: the magnitude of motion in lateral, anterior-posterior (AP), and superior-inferior (SI) direction of ten respiratory phases, respectively.

**Table 4:** GTV centriod movement by different positional and attachment status.

Tumor location	Magnitude of solitary tumor (mean ± SD mm) *n* = 14				Magnitude of attached tumor (mean ± SD mm) *n* = 30			
	*n*	Later	AP	SI	*n*	Later	AP	SI
Upper level	9	0.9 ± 0.3	1.5 ± 1.2	2.3 ± 2.2	20	0.9 ± 0.5	1.2 ± 0.7	1.5 ± 1.7
Middle level	1	2.6	5.2	11.7	7	1.5 ± 0.7	2.1 ± 0.9	2.2 ± 1.3
Lower level	4*	2.5 ± 1.9	2.7 ± 1.5	10 ± 4.8	3	1.5 ± 0.9	1.5 ± 0.7	3.0 ± 1.9

*This value included 3 cases whose lesions attached to diaphragm.

Upper level included right upper lobe, anterior, and apicoposterior segment in left upper lobe. Middle level included right middle lobe and lingular bronchus in left lobe. Lower level included left and right lower lobes.

SD: standard deviation; AP: anterior-posterior direction, and SI: superior-inferior direction.
